# “Amr Sign”: A Case-Control Study Evaluating the Diagnostic Value of a New Clinical Sign in the Diagnosis of Acute Appendicitis

**DOI:** 10.7759/cureus.70222

**Published:** 2024-09-25

**Authors:** Amr Mohamed F Aboulwafa, Ali A Aboulwafa, Khalil Ahmad, Mazin Abouzour, Amira Khairallah

**Affiliations:** 1 Surgery, Westbay Medicare Hospital, Doha, QAT; 2 Medicine, Weill Cornell Medicine - Qatar, Doha, QAT; 3 Statistics, Government Graduate College, Asghar Mall Rawalpindi, Rawalpindi, PAK; 4 Healthcare Professions, Ministry of Public Health, Doha, QAT

**Keywords:** acute appendicitis, alvarado score, amr sign, diagnosis, diagnostic reliability

## Abstract

Introduction

Acute appendicitis, a prevalent cause of acute abdominal pain and a common indication for emergency surgery, presents a diagnostic challenge due to its diverse clinical presentation and variability in appendix location and symptoms. Traditional diagnostic approaches, including physical examination, clinical scoring systems, and imaging techniques, have limitations. This study introduces the "Amr sign," a new diagnostic indicator characterized by sudden reflex hyperextension of the neck upon palpation of the right iliac fossa, which is hypothesized to reflect local peritoneal irritation from an inflamed appendix.

Methods

We conducted a prospective observational study at Alwakra Hospital from November 2016 to January 2019 that included 195 patients aged 15 years and older with right iliac fossa pain and an Alvarado score of four or more. The "Amr sign" was evaluated alongside standard clinical examination and imaging results. Diagnostic accuracy was assessed via histopathological confirmation, which is the gold standard.

Results

The "Amr sign" had a sensitivity of 68.6% and a specificity of 67.3%. The positive predictive value (PPV) was 89%, whereas the negative predictive value (NPV) was 45%. Receiver operating characteristic (ROC) analysis revealed an area under the curve (AUC) of 0.679 for the "Amr sign" compared with 0.622 for the other imaging modalities, suggesting comparable diagnostic performance. The "Amr sign" demonstrated a higher likelihood ratio of a positive test (2.3) and a lower likelihood ratio of a negative test (0.46).

Conclusion

The "Amr sign" is a promising diagnostic tool for acute appendicitis, offering a simple, reliable, and quick test that can be performed by less experienced physicians. While its sensitivity and specificity are moderate, its high PPV suggests that it is particularly useful in confirming appendicitis. Further research and validation are needed to fully establish its role in clinical practice.

## Introduction

Acute appendicitis is one of the most common causes of acute abdomen and the leading indication for emergency abdominal surgery worldwide. In the USA, the annual incidence is 93 per 100,000 people, making it the most frequent acute surgical emergency in the country [1]. A higher incidence has been reported in European populations, with 132 cases per 100,000 in Spain [2]. However, reports from Denmark and the UK have noted a decline in the incidence of acute appendicitis, though no specific cause has been proposed for this trend [3]. Although the management of acute appendicitis is standardized, the diagnosis of appendicitis remains a clinical challenge for physicians. Most medical students and residents learn how to diagnose a hernia within one to two teaching sessions, but it takes months of experience to master the skill of diagnosing the causes of right iliac fossa (RIF) pain. This is due to the multiplicity of pathologies in the RIF, which can manifest themselves in the variability in appendix position, time of presentation, pain tolerance, and the lack of one specific accurate and sensitive clinical sign. For these reasons, surgeons have traditionally accepted a 20% rate of false positive diagnoses of acute appendicitis, leading to a significant number of unnecessary surgeries, particularly in females, to minimize morbidities associated with a missed diagnosis [4]. The false positive diagnosis rate of appendicitis in clinical practice is particularly high in females, with reports reaching up to 42% [5]. While both genders pose diagnostic challenges, women often require additional imaging, such as ultrasonography, to rule out gynecological and obstetric causes, whereas men rely more on repeated physical examinations [6,7].

Since repeated physical examination is the gold standard of diagnosis, and to avoid subjective methods of diagnosis, a number of clinical scoring systems have been devised [8]. They gained popularity as they rendered the diagnosis of appendicitis more objective, though not particularly perfect. Eventually, the scoring systems lost popularity, and we returned to the era of clinical judgment. Although initial reports and studies on the diagnostic power of CT and abdominal ultrasound (AUS) were not promising at the beginning of the third millennium [9], trials of the use of technetium 99 labeled WBCs in the diagnosis of acute appendicitis were introduced, but again, the results were not encouraging, the cost was high, and the feasibility of such techniques in hospital was limited [10]. During the last three decades, with the advent of high-resolution ultrasound and the rise of the learning curve among radiologists, the diagnostic benefit of ultrasound and CT scans has been confirmed, and their use has become almost standard practice in the evaluation of acute appendicitis [11-13].

Several studies have emphasized the efficacy of adding ultrasound to clinical examination or the Alvarado score [14-16]. A careful history and the signs elicited will, in most cases, lead to a diagnosis. However, the diagnostic challenge lies in a significant portion of patients who present with vague symptoms or incomplete clinical signs. At this point, it is up to the physician’s experience to reach a conclusion with respect to operation, observation, investigation, or discharge. For this reason, we present a new sign that we believe has far greater sensitivity and specificity for diagnosing and excluding acute appendicitis than any other clinical sign. We present the “Amr sign,” which is simply a sudden reflex hyperextension of the neck upon palpation of the RIF in patients with acute appendicitis. The explanation for such a jerky reflex is probably the presence of some degree of local peritonitis over the inflamed appendix. This explanation is augmented by the fact that the sign was difficult to elicit in the retrocecal position of the inflamed appendix. It is a very simple, reliable, quick test that is safe to conduct and can be easily performed by even the least experienced physicians.

## Materials and methods

During the period from November 2016 to January 2019, we prospectively examined 440 patients above the age of 15 who presented to the Accident and Emergency Department of Alwakra Hospital in the State of Qatar with a complaint of RIF pain. A total of 440 patients were initially examined by the emergency physician, and those who had an Alvarado score of four or more were referred to the surgeon with an initial diagnosis of suspected acute appendicitis. A total of 195 patients were referred and included in the study. To guarantee the consistency of the technique, the eliciting of the sign was performed by the same physician and medical student. The sign was considered positive if sudden hyperextension of the neck occurred in response to pressure on the RIF and negative if no neck movement occurred.

Patient selection

Patients of both sexes, all above the age of 15 years, presenting with RIF pain and an Alvarado score of four or more during the period from November 2016 to January 2019, were included in the study (standard examination plus the "Amr sign"). In addition to the Alvarado score and RIF pain, other clinical factors such as fever, leukocytosis, and localized tenderness were also considered as part of the standard clinical assessment (standard examination plus the "Amr sign").

Exclusion criteria

Patients younger than 15 years of age, those with an Alvarado score below four, and those with a history of appendectomy were excluded from the study. Any of the causes of abdominal pain, such as gastrointestinal disorders, gynecological conditions, or urinary tract infections, were still included in the study to test the specificity of "Amr sign" and its ability to pick up only appendicitis among all patients with RIF pain.

Ethics

A copy of the project was sent to the medical ethics committee and approved.

The study was conducted as an observational study, where the results of the "Amr sign" were not considered a factor in determining the decision to operate or observe.

Blinding

The physician performing the clinical test ("Amr sign") performed a physical examination and recorded whether the test was positive or negative. He was not involved in the patient’s treatment.

The final confirmation of the diagnosis of acute appendicitis was made via histopathological examination of the retrieved appendix. Patients who were discharged without surgery due to a lack of symptoms and signs were considered negative for appendicitis. They were included in the study to test the negative predictive value (NPV) of this sign. At the end of the study, the results were collected and compared against other known signs of appendicitis.

Statistical analysis

The data were compiled, checked, and analyzed using the Statistical Package for Social Sciences (IBM Corp., Armonk, NY) version 26. For each variable and all demographic variables, descriptive statistics (i.e., frequencies, percentages, and measures of central tendency and where applicable) were generated. A chi-square test was implemented to test the significance of the associations of "Amr sign," CT scan, and ultrasound, with other attributes at the 5% level of significance, with the CT scan results and between male and female students. ROC analysis was performed to test the sensitivity and specificity of the "Amr sign" and imaging modalities.

## Results

The demographic distribution of patients diagnosed with acute appendicitis is detailed in Table 1. The majority of the cases (64.0%) occurred in the age group of 15-30 years, followed by 31-50 years (33.8%), and a small proportion (2.2%) in patients older than 50 years.

**Table 1:** Demographics of patients with acute appendicitis

Variables/categories	n	%
Age groups		
15-30 years	87	64.0%
31-50 years	46	33.8%
>50 years	3	2.2%
Gender		
Female	32	16.4%
Male	163	83.6%

**Figure 1:**
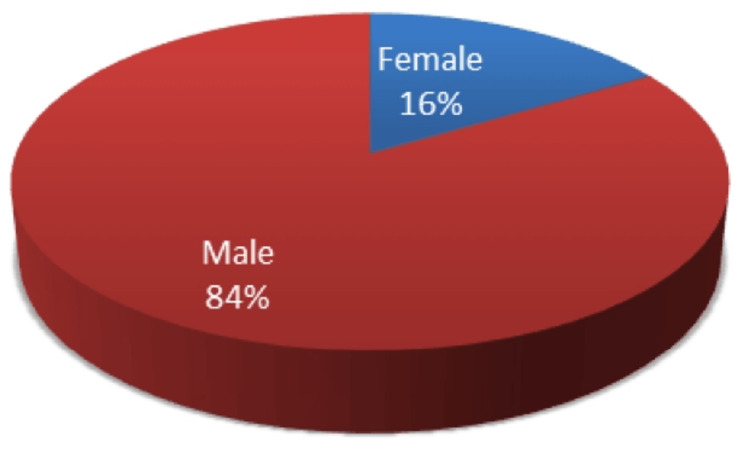
Gender distribution of the patients of acute appendicitis included in our study

Gender distribution indicated a significantly higher incidence in males, who comprised 83.6% of the cases, compared to females at 16.4% (Figure 1).

The analysis of the frequency distribution of patients with acute appendicitis, as presented in Table 2, reveals several key clinical indicators associated with the condition. Among the 195 patients studied, 59.0% (n = 115) experienced vomiting, and 30.3% (n = 59) presented with fever. A significant majority, 97.4% (n = 190), exhibited tenderness in the RIF, while 66.2% (n = 129) had positive cough signs. Regarding patient outcomes, 77.9% (n = 152) underwent surgical intervention, and 22.1% (n = 43) were observed without immediate surgery. Rebound tenderness and a neutrophil shift to the left were noted in 55.4% (n = 108) and 56.4% (n = 110) of patients, respectively. Rovsing’s sign was positive in 44.1% (n = 86) of cases, while 52.3% (n = 102) reported migrating pain. These findings underscore the prevalence of these symptoms in diagnosing acute appendicitis, with tenderness in RIF and cough signs being particularly prominent indicators. The statistical analysis supports these observations, with a 95% confidence interval (CI) demonstrating the robustness of the results, although specific p-values were not provided for these individual attributes.

**Table 2:** Conditions of patients with acute appendicitis RIF, right iliac fossa

Attributes/categories	n	%
Vomiting		
No	80	41.0 %
Yes	115	59.0 %
Fever		
No	136	69.7 %
Yes	59	30.3 %
Tender RIF		
Negative	5	2.6 %
Positive	190	97.4 %
Cough signs		
Negative	66	33.8%
Positive	129	66.2%
Disposition		
Total admitted	195	100 %
Operated	152	77.9 %
Observed	43	22.1 %
Rebound tenderness		
Negative	87	44.6 %
Positive	108	55.4 %
Neutrophil shift to left		
Negative	85	43.6 %
Positive	110	56.4 %
Rovsing’s sign		
Negative	109	55.9%
Positive	86	44.1%
Migrating pain		
Negative	93	47.7%
Positive	102	52.3%

The results presented in Table 3 provide a detailed comparison of the "Amr sign" and other clinical findings among patients suspected of having acute appendicitis. The "Amr sign" was positive in 58.5% of the patients (n = 114) and negative in 41.5% (n = 81), indicating a relatively high frequency of this sign among those diagnosed with appendicitis. Of the 195 patients admitted, 77.9% (n = 152) underwent surgery, and 22.1% (n = 43) were observed. Histopathological examination confirmed acute appendicitis in 71.8% (n = 140) of cases, while 8.2% (n = 16) had negative results, and in 20% (n = 39) of the cases, the histopathology was not performed due to discharge of the patient without surgery. The total number of negative histopathology and discharged cases without surgery is 55 (28.2 %). Notably, Rovsing’s sign was positive in 44.1% (n = 86) of the patients, while imaging findings varied, with 48.7% (n = 95) showing positive results on imaging modalities (ultrasound or CT) or negative results in 15.9% (n = 31), while 5.1% (n = 10) were inconclusive, and 30.3% (n = 59) were those where neither ultrasound nor CT was performed. The high positive rate of the "Amr sign" compared to Rovsing’s sign (44.1%) and imaging results suggests its potential utility in diagnosing acute appendicitis. Further statistical analysis with a 95% CI and p-value would be necessary to determine the precise sensitivity and specificity of the "Amr sign" in comparison to other diagnostic methods.

**Table 3:** "Amr sign" and other clinical findings of patients with acute appendicitis

Attributes/categories	n	%
"Amr sign"		
Negative	81	41.5%
Positive	114	58.5%
Disposition		
Total admitted	195	100%
Operated	152	77.9%
Observed	43	22.1%
Histopathology		
Positive	140	71.8%
Negative	16	8.2 %
Discharged no surgery	39	20.0%
Overall negative	55	28.2 %
Rovsing’s sign		
Negative	109	55.9%
Positive	86	44.1%
Image modalities		
Negative	31	15.9%
Positive	95	48.7%
Inconclusive	10	5.1%
Not done	59	30.3%

The association of the "Amr sign" with various demographic and clinical attributes in patients with acute appendicitis was analyzed, revealing several statistically significant findings (Table 4). Age group showed a significant association with the "Amr sign" (χ² = 4.418, p = 0.041), with a higher prevalence of a positive "Amr sign" in the 15-30 years age group (58.1%) compared to the negative group (74.0%). Gender, vomiting, fever, tender RIF, cough signs, previous admission, rebound tenderness, neutrophil shift to the left, Rovsing’s sign, and migrating pain were not significantly associated with the "Amr sign" (p > 0.05). However, histopathology results showed a highly significant association (χ² = 20.892, p < 0.001), with 84.2% of patients with a positive "Amr sign" having confirmed acute appendicitis, compared to 54.3% in the negative group. The disposition variable approached significance (χ² = 3.244, p = 0.072), suggesting a trend toward more frequent surgical intervention in patients with a positive "Amr sign" (82.5%). These results highlight the potential diagnostic value of the "Amr sign" in acute appendicitis, particularly when considered alongside histopathological findings.

**Table 4:** Testing significance of the association of "Amr sign" with other demographic and clinical attributes of patients with acute appendicitis Highly significant if p < 0.01, significant if p < 0.05, and nonsignificant if p > 0.05. RIF, right iliac fossa

Variables/categories		"Amr sign"				Chi-square	p-value
		Negative		Positive			
		n	%	n	%		
Age groups	15-30 years	37	74.0%	50	58.1%	4.418	0.041
	31-50 years	13	26.0%	33	38.4%		
	>50 years	0	0.0%	3	3.5%		
Gender	Female	15	18.5%	17	14.9%	0.449	0.503
	Male	66	81.5%	97	85.1%		
Vomiting	No	30	37.0%	50	43.9%	0.991	0.340
	Yes	51	63.0%	64	56.1%		
Fever	No	54	66.7%	82	71.9%	0.622	0.430
	Yes	27	33.3%	32	28.1%		
Tender RIF	Negative	9	11.1%	17	14.9%	0.592	0.442
	Positive	72	88.9%	97	85.1%		
Cough signs	Negative	30	37.0%	36	31.6%	0.630	0.427
	Positive	51	63.0%	78	68.4%		
Disposition	Operated	58	71.6%	94	82.5%	3.244	0.072
	Observed	23	28.4%	20	17.5%		
Histopathology	Negative	37	45.7%	18	15.8%	20.892	0.000
	Positive	44	54.3%	96	84.2%		
Previous admission	No	49	60.5%	73	64.0%	0.254	0.615
	Yes	32	39.5%	41	36.0%		
Rebound tenderness	Negative	36	44.4%	51	44.7%	0.002	0.968
	Positive	45	55.6%	63	55.3%		
Neutrophil shift to left	Negative	33	40.7%	52	45.6%	0.457	0.499
	Positive	48	59.3%	62	54.4%		
Rovsing’s sign	Negative	44	54.3%	65	57.0%	0.140	0.709
	Positive	37	45.7%	49	43.0%		
Migrating pain	Negative	36	44.4%	57	50.0%	0.586	0.444
	Positive	45	55.6%	57	50.0%		

The diagnostic accuracy of the "Amr sign" and imaging modalities in predicting acute appendicitis was assessed using receiver operating characteristic (ROC) analysis, as shown in Table 5. The area under the curve (AUC) for the "Amr sign" was 0.679 (p < 0.001), with a 95% CI ranging from 0.606 to 0.753. This indicates a moderate level of diagnostic accuracy, as values closer to 1.0 suggest higher accuracy. In comparison, imaging modalities yielded a lower AUC of 0.622 (p = 0.017) with a 95% CI of 0.522 to 0.722, indicating a statistically significant but less accurate performance compared to the "Amr sign." These results suggest that the "Amr sign" may offer a more reliable diagnostic tool for acute appendicitis than imaging modalities, although neither approach achieves perfect accuracy.

**Table 5:** Area under the receiver operating characteristic curve The test result variables, "Amr sign" and imaging modalities, have at least one tie between the positive actual state group and the negative actual state group. Null hypothesis: true area = 0.5

Test result variable	Area	Standard error	p-value	95% confidence interval	
				Lower bound	Upper bound
"Amr sign"	0.679	0.038	0.000	0.606	0.753
Imaging modalities	0.622	0.051	0.017	0.522	0.722

In the evaluation of the diagnostic performance of the "Amr sign" compared to imaging modalities, the ROC analysis reveals that the "Amr sign" has a Gini index of 0.358 and a corresponding Kolmogorov-Smirnov (K-S) statistic of 0.358, with a maximum K-S cutoff at 0.50 (Table 6). In comparison, imaging modalities show a slightly lower Gini index of 0.244 but a higher K-S statistic of 0.386, also with a maximum K-S cutoff at 0.50. The p-values associated with these metrics indicate the statistical significance of the observed differences. These results suggest that while both diagnostic tools provide valuable information, the "Amr sign" exhibits a slightly better discriminatory power, as reflected by its Gini index, though the K-S statistic for imaging modalities is marginally higher, indicating a potentially stronger separation between true positive and false positive rates at the given cutoff.

**Table 6:** Classifier evaluation metrics

Test result variable	Gini index	K-S statistics	
		Max K-S	Cutoff
"Amr sign"	0.358	0.358	0.50
Imaging modalities	0.244	0.386	0.50

**Figure 2:**
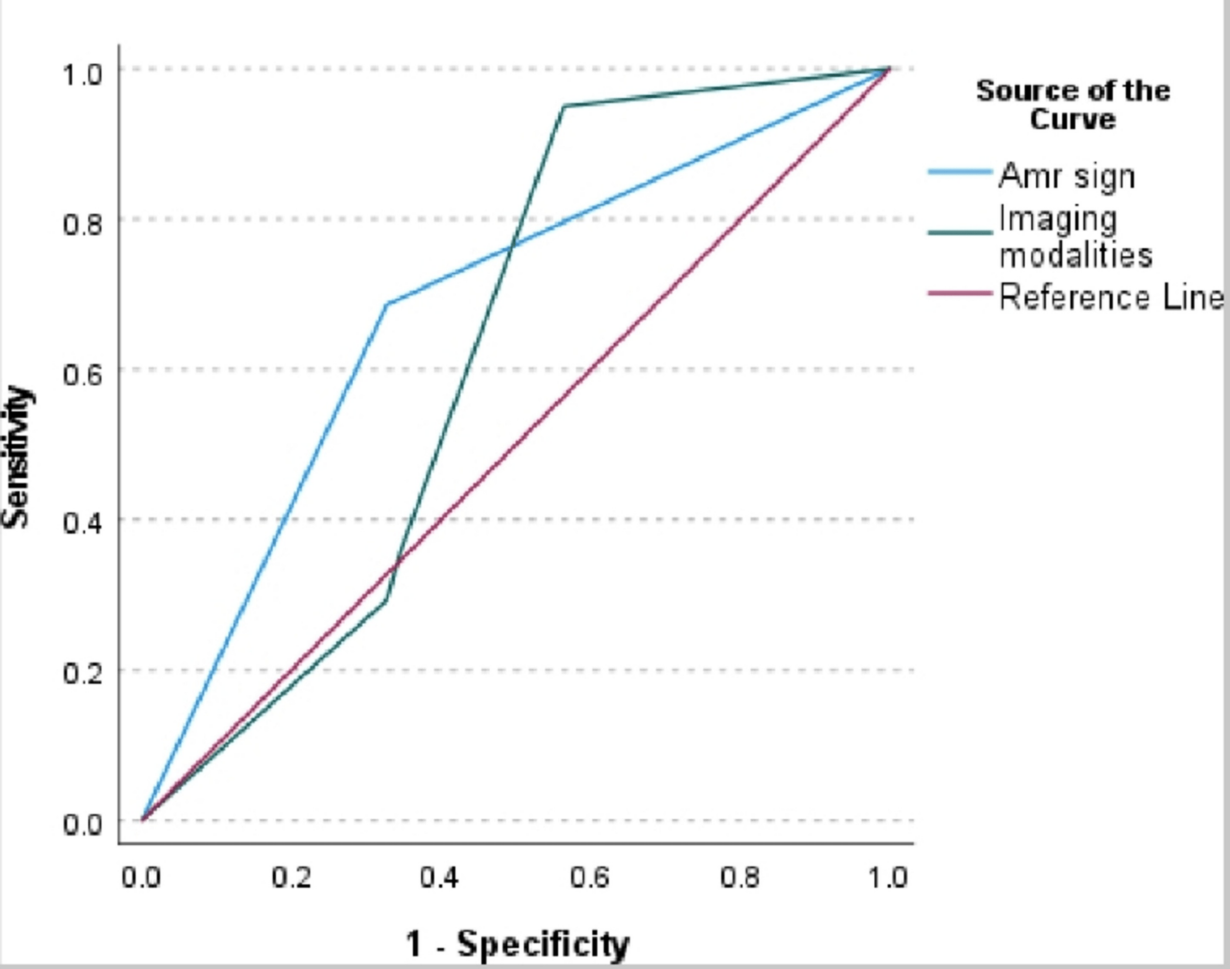
Receiver operating characteristic curve analysis

The results of the ROC analysis presented in Figure 2 indicate that the "Amr sign" when compared to imaging modalities, has a statistically insignificant difference in diagnostic performance for acute appendicitis.

The AUC difference between the "Amr sign" sign and imaging modalities was found to be 0.849, with a standard error difference of 0.057 and a 95% CI ranging from -0.075 to 0.190 (Table 7). The z-value of 0.396 and the corresponding p-value of 0.298 suggest that the observed difference is not statistically significant, failing to reject the null hypothesis that the true area difference is zero. This implies that the "Amr sign" performs comparably to imaging modalities in diagnosing acute appendicitis but without significant superiority.

**Table 7:** Paired-sample area difference under the receiver operating characteristic curves Highly significant if p < 0.01, significant if p < 0.05, and nonsignificant if p > 0.05. Null hypothesis: true area difference = 0 AUC, area under the curve

Test result pair	Asymptotic		AUC difference	Standard error difference	95% confidence interval	
	z	p-value			Lower bound	Upper bound
"Amr sign" - imaging modalities	0.849	0.396	0.057	0.298	-0.075	0.190

The diagnostic performance of the "Amr sign" in detecting acute appendicitis was evaluated by comparing its results against histopathological findings. According to Table 8, the "Amr sign" demonstrated a sensitivity of 68.6% and specificity of 67.3% at a threshold of 0.50. In contrast, imaging modalities display a higher sensitivity of 95.0% but a lower specificity of 43.6% at the same threshold. The ROC analysis reveals that while the "Amr sign" offers a balanced sensitivity and specificity, imaging modalities provide greater sensitivity but at the expense of specificity. The positive predictive value (PPV) of the "Amr sign" was 89%, indicating a high likelihood that patients with a positive "Amr sign" truly have acute appendicitis. Conversely, the NPV was 45%, suggesting a lower likelihood of correctly identifying patients who do not have appendicitis when the test result is negative. The likelihood ratio of a positive test was calculated to be 2.3, indicating that a positive "Amr sign" is 2.3 times more likely to be associated with acute appendicitis than a negative sign. Conversely, the likelihood ratio of a negative test was 0.46, showing that a negative "Amr sign" is less effective in ruling out appendicitis. Overall, while the "Amr sign" has a reasonable PPV, its relatively low sensitivity and NPV suggest that it should be used in conjunction with other diagnostic methods rather than as a standalone test.

**Table 8:** Coordinates of the receiver operating characteristic curve

Test result variable	Positive if greater than or equal to	Sensitivity	1 - Specificity
"Amr sign"	-1.00	1.000	1.000
	0.50	0.686	0.327
	2.00	0.000	0.000
Imaging modalities	-1.00	1.000	1.000
	0.50	0.950	0.564
	1.50	0.357	0.345
	2.50	0.293	0.327
	4.00	0.000	0.000

Table 9 compares the positive and negative likelihood ratios of "Amr sign," RIF tenderness, and leukocytosis in order to show which of the three signs has more reliability to rule in or out acute appendicitis.

**Table 9:** Results of likelihood ratio test RIF, right iliac fossa

Test	Positive likelihood ratio	Trust when test is positive	Negative likelihood ratio	Trust when test is negative (reliability)
"Amr sign"	2.3	Small	0.46	Small
RIF tenderness	1	Minimal	0.4	Small
Leukocytosis	1.3	Minimal	0.24	Small

## Discussion

One of the strengths of this study is the diversity of the patient population. Qatar, a multinational country, is home to many ethnic groups from different nationalities [17]. The 195 patients included in this study represented 25 nationalities and seven ethnic groups. This diversity enhances the credibility of our study, indicating that the findings are likely to be applicable to populations globally. 

In this study, the majority of the cases (64.0%) occurred in the age group of 15-30 years. Our findings are in accordance with previous studies reporting that the majority of the cases (43.3 and 47%) were in the age group of 15-30 years [18,19]. Interestingly, gender distribution revealed a significantly higher incidence in males (83.6%) than in females (16.4%) cases. These findings suggest that acute appendicitis predominantly affects younger individuals, particularly males, which aligns with existing epidemiological data on the condition [18,19]. Our findings underscore the importance of considering demographic factors when diagnosing and managing acute appendicitis in clinical practice.

Our study revealed a strong PPV of the "Amr sign" when tested separately to diagnose appendicitis; although it did not show superiority over imaging, it did show a higher specificity and sensitivity when compared to each clinical sign of the Alvarado score individually. Previously, it has been reported in a recent meta-analysis that the Alvarado score demonstrated low combined sensitivity and specificity in diagnosing acute appendicitis [20]. When the "Amr sign" is compared with the two most important and pivotal signs in the Alvarado score - RIF tenderness and leukocytosis (each weighted at two points instead of one) - RIF tenderness had very high sensitivity (98.5%) but very low specificity (3.7%) compared to the "Amr sign" (68% sensitivity) and (70% specificity). Hence, RIF pain alone, although highly sensitive for appendicitis, does not help in ruling out the disease when the sign is positive simply because a highly sensitive test would be very useful in ruling out a disease when the test is negative. Since it has a very low specificity (3.7%), a positive RIF pain could indicate appendicitis, among many other causes of RIF pain. Thus, a higher specificity test, such as the "Amr sign" (70%), would be more useful in confirming the disease when the test is positive, and to a lesser extent, it might be fairly helpful in ruling out the disease when the sign is negative. Similarly, leukocytosis alone had a sensitivity of 93.6% but a specificity of only 26.4%, meaning it also does little to confirm appendicitis even when the white cell count is elevated. This is further confirmed by the positive likelihood ratio of only 1.3 and the negative likelihood ratio of 0.24, which suggests that leukocytosis, as a standalone test, has minimal importance in definitively ruling in or out the disease.

Our results further revealed that the "Amr sign" is superior to RIF tenderness and leukocytosis when positive. Our results are consistent with those of Iftikhar et al., who reported that the Pediatric Appendicitis Score (PAS) technique is superior to the Alvarado score in diagnosing acute appendicitis in the pediatric population, as indicated by higher diagnostic accuracy values [21]. Considering that when a test’s reliability is minimal, it cannot be used alone in the diagnosis or exclusion of a disease, while a test with “low” reliability can still be effective when combined with other tests. Thus, a more reliable test in diagnosing appendicitis may increase diagnosis certainty, though it remains less reliable when negative for ruling out appendicitis. This leads us to conclude that incorporating the "Amr sign" into the Alvarado score clinical criteria can significantly improve the PPV for diagnosing acute appendicitis. As a result, this combination may reduce the need for expensive, time-consuming, and costly imaging studies to confirm the diagnosis.

Limitations

One limitation of this study is that the "Amr sign," despite its promising PPV, was only compared to the two most pivotal clinical signs of the Alvarado score - RIF tenderness and leukocytosis. A more comprehensive comparison with other established diagnostic criteria and imaging modalities would provide a clearer picture of its diagnostic utility. Additionally, the study did not assess the performance of the "Amr sign" across different age groups or gender distributions, which could affect its generalizability. Lastly, while the study highlights the potential of the "Amr sign" to reduce reliance on imaging, it does not address how the inclusion of this sign might impact the overall clinical decision-making process in real-world settings where resources vary.

## Conclusions

In this study, the "Amr sign" has been evaluated as a diagnostic tool for acute appendicitis, showing moderate sensitivity (68.6%) and specificity (67.3%). Although its diagnostic performance is not superior to imaging modalities, the sign's high PPV (89%) and ease of use make it a valuable addition to the diagnostic arsenal. The ROC analysis indicated that the "Amr sign" provides comparable diagnostic accuracy to imaging techniques, with an AUC of 0.679. While it does not achieve perfect accuracy, its simple application and reliability in detecting appendicitis justify its consideration as a supplementary diagnostic method. The relatively low NPV (45%) of the "Amr sign" highlights the importance of using it alongside other diagnostic methods, such as the Alvarado score or imaging, when ruling out appendicitis. On its own, the "Amr sign" is less reliable when negative. However, when positive, it improves diagnostic certainty. This leads us to conclude that incorporating the "Amr sign" into the Alvarado score significantly enhances the PPV of current clinical and imaging methods used for diagnosing acute appendicitis.
